# Unraveling the role of lactylation in cell death and correlative diseases: a comprehensive review

**DOI:** 10.3389/fcell.2026.1874067

**Published:** 2026-07-01

**Authors:** Bowen Zhao, Yue Liu, Xinpei Shen, Yunxue Zhang, Qihang Cang, Yuan Fan, Yanting Wang

**Affiliations:** 1 Stomatological College of Nanjing Medical University, Nanjing, China; 2 Department of Oral Mucosal Diseases, The Affiliated Stomatological Hospital of Nanjing Medical University, Nanjing, China; 3 State Key Laboratory Cultivation Base of Research, Prevention and Treatment for Oral Diseases, Nanjing, China; 4 Jiangsu Province Engineering Research Center of Stomatological Translational Medicine, Nanjing, China

**Keywords:** lactylation, apoptosis, autophagy, pyroptosis, ferroptosis, cuproptosis

## Abstract

Lactylation is a newly identified post-translational modification and an important biological function of lactate. Numerous pathological disorders, including cancer and autoimmune and inflammatory diseases, are regulated by lactylation. Additionally, lactylation controls several physiological processes in cells, such as the production of proteins, cytokines, metabolism, and several forms of cell death. Cell death is responsible for tissue homeostasis, immune responses, tissue repair and tissue regeneration. Recent studies have revealed a close association between lactylation and cell death, underscoring their significance in the development and course of illness. The regulation of different types of cell death by lactylation involves the modulation of key cell death proteins. Therefore, clarifying the precise function of lactylation and related cell death mechanisms is essential for targeted disease therapies. Here, we provide an overview of how lactylation regulates cellular signaling in cells undergoing apoptosis, autophagy, pyroptosis, ferroptosis, and cuproptosis. Lactylation intervention to control cell death may offer new therapeutic insights for a number of human diseases.

## 1 Introduction

The biological mechanisms known as protein post-translational modifications (PTMs) are vital for controlling the structure, activity, and interactions of proteins (Millán-Zambrano et al., 2022). Abnormalities in PTMs can lead to altered protein activity and the loss of biological function, resulting in cellular dysfunction and contributing to the onset and progression of various diseases. Recently, a novel type of PTM-lactylation has been discovered, offering new insights into the functional roles of lactate (Zhang et al., 2019). Otto Warburg reported that even under normoxic conditions, rapidly proliferating tumor cells preferentially utilize glycolysis to generate energy, which causes lactate to build up both inside and outside the cell (Vaupel and Multhoff, 2021). This process is referred to as aerobic glycolysis. The reason why tumor cells favor this relatively inefficient metabolic pathway over more energy-efficient oxidative phosphorylation remains unclear. The discovery of lactylation has revealed the broader significance of lactate in biological processes. Increasing evidence indicates that lactylation is intimately linked to numerous essential cellular physiological functions, such as gene expression regulation, signal transduction, metabolic control, immunological responses, and cell death. Moreover, lactylation has been implicated in the development of various diseases, such as cancer, neurological and cardiovascular disorders, and inflammation.

Cell death is a basic physiological process that plays both physiological and pathological roles. Cell death is involved not only in embryonic development, organ maintenance, and aging but also in immune responses and the regulation of autoimmunity. Cell death can be triggered by programmed genetic mechanisms, such as apoptosis, autophagy, and pyroptosis, or can result from metabolic dysregulation, such as ferroptosis (Newton et al., 2024; Zhou et al., 2024). Lactylation is implicated in several types of cell death, such as apoptosis, autophagy, pyroptosis, and ferroptosis, according to recent research.

Here, we look into how lactylation and cell death interact in correlative diseases. Specifically, we summarize the latest techniques for detecting lactylation, discuss the chemical characteristics of lactylation reactions and the key enzymes involved, and analyze its role in five different forms of cell death. Furthermore, we evaluated the possibility of using lactylation-mediated cell death as a therapeutic target to treat correlative diseases.

## 2 Lactylation: a novel protein post-translational modification

### 2.1 Detection techniques for lysine lactylation

The discovery of lysine lactylation has been largely driven by advances in analytical technologies. Mass spectrometry (MS) is now the most useful approach for proteomic analyses of PTMs thanks to the development of highly sensitive high-resolution mass spectrometers, sophisticated search algorithms, and powerful bioinformatics tools (Brandi et al., 2022). High-performance liquid chromatography-tandem mass spectrometry (HPLC-MS) was used to identify histone lactylation for the first time. Three proteolytic peptides derived from trypsin-digested core histones of human MCF-7 cells showed a mass shift of 72.021 Da at lysine residues (Zhang et al., 2019). However, conventional PTM identification relies on fixed tandem MS (MS/MS) fragment ions, and inaccuracies in spectral matching frequently result in false-positive assignments. Wan et al. reported that in tandem MS analysis, the formation of cyclic immonium ions derived from lactylated lysine can reliably facilitate the profiling of lysine lactylation (Kla)-containing proteins. Notably, Cyclm ions in Kla exhibit a strong affinity for Kla spectra. Wan et al. proposed incorporating this ion as a criterion for Kla identification (Wan et al., 2022).

In addition to improvements in conventional HPLC-MS, the accumulation of experimental data and the iterative refinement of algorithmic models have facilitated the increasing application of artificial intelligence (AI) in the prediction and analysis of lactylation. AI can substantially reduce detection time and cost, thereby enabling large-scale identification and characterization of lactylation. FSL-Kla represents the first model and web server designed to forecast Kla sites utilizing a learning-based multifeature fusion system, which is freely accessible online (http://kla.zbiolab.cn/). FSL-Kla is a state-of-the-art tool for Kla site profiling and a resource for producing candidates for further experimental validation by combining eight sequence-based features with three structure-based features (Jiang et al., 2021). Subsequently, the first hybrid deep neural network and web server based on a convolutional neural network-bidirectional gated recurrent units-attention mechanism, DeepKla, was developed by Lv et al. for the prediction of Kla sites in rice (Lv et al., 2022). Both of these models present two major limitations: first, the small dataset size restricts the amount of available information, and second, they rely on extensive manual hyperparameter tuning to achieve optimal performance. To address these issues, Auto-Kla was developed, which employs automated machine learning techniques to train a natural language processing model on a dataset consisting of 2,375 Kla sites identified in stomach cancer AGS cells, thereby allowing for accurate prediction of Kla sites. The Auto-Kla web server is freely available at http://tubic.org/Kla (Lai and Gao, 2023). In addition, new tools such as PBertKla and A/EBFF-Kla have been continuously developed, which help to overcome the obstacles associated with identifying Kla sites and thereby facilitate deeper exploration of their critical roles in the regulation of cellular functions (Yang et al., 2024; Lai et al., 2025).

The advancement of detection techniques for lysine lactylation has also relied heavily on the development and optimization of specific antibodies, including both pan-Kla antibodies and site-specific antibodies. In 2019, Zhang et al. introduced an immunoprecipitation approach based on a pan-Kla antibody combined with MS/MS to identify lactylated target proteins and modification sites in cells (Zhang et al., 2019). Site-specific antibodies can be integrated with high-throughput sequencing technologies such as ChIP-seq and CUT&Tag. ChIP-seq, employing site-specific antibodies, enables precise genome-wide capture of lactylation-enriched regions, thereby revealing its direct association with gene transcriptional regulation. This approach is often integrated with multi-omics data including RNA-seq to screen for downstream target genes directly regulated by lactylation, a strategy that has been successfully validated in a range of diseases (Liu J. et al., 2025; Zhu Y. et al., 2025; Cheng et al., 2026).

### 2.2 Histone lysine lactylation and nonhistone lysine lactylation

Chromatin is a linear complex structure within the interphase nucleus that is composed of DNA, histones, nonhistone proteins, and a small amount of RNA. Its fundamental unit is the nucleosome, which is made up of DNA wrapped around a core of four central histones (H3, H4, H2A, and H2B). The N-terminal tails of histones undergo a wide variety of PTMs, which regulate processes like transcription, replication, and DNA repair by modulating the winding and unwinding of DNA (Dai et al., 2022). To date, many different types of PTMs have been identified on histones (Wu et al., 2023). These modifications can alter the shape of nucleosomes by modulating the positive and negative charges of histone residues, thereby influencing the loosening or tightening of the DNA helix and subsequently regulating gene expression (Prakash and Fournier, 2018). Many histone lactylation sites have been found since Yingming Zhao at the University of Chicago originally reported the discovery of histone lactylation in 2019 (Zhang et al., 2019). Numerous histone lactylation sites have been found thus far, among which H3 lysine 18 lactylation (H3K18la), H3 lysine 9 lactylation (H3K9la), H3 lysine 14 lactylation (H3K14la), and H3 lysine 56 lactylation (H3K56la) are strongly linked to cell death (Li et al., 2022). H3K18la is particularly regarded as a key marker closely associated with multiple forms of cell death (Chu et al., 2021; Galle et al., 2022; Li W. et al., 2024).

Lactylation occurs on both histones and nonhistone proteins, significantly increasing protein diversity. With the advancement of detection technologies such as liquid chromatography-tandem mass spectrometry, many nonhistone lactylation sites have been identified across various cellular structures, including the cell membrane, cytoplasm, nucleus, mitochondria, and ribosomes (Luo et al., 2022). Yang et al. discovered 2,375 lactylation sites in 1,014 proteins from cancer cells, emphasizing the pervasive occurrence of lactylation (Yang et al., 2022). Nonhistone modifications represent an important mechanism through which lactylation regulates cellular aging and death, as it can alter protein activity, affect subcellular localization, and influence protein-protein interactions.

### 2.3 Classification of lactylation

There are now two recognized lactylation mechanisms: enzymatic and non-enzymatic. A common substrate for both routes, lactate, has distinct chiral forms. D-lactate takes part in the non-enzymatic phase of lactylation, whereas L-lactate is involved in the enzymatic process. There are two different routes involved in enzymatic lactylation. L-lactate is converted to L-lactyl-CoA, a direct substrate for lactylation, in the first pathway, which was suggested since lactylation and acetylation are related processes. Writer enzymes facilitate the transfer of the lactyl group to lysine residues on target proteins by L-lactyl-CoA (Zhang et al., 2019). Alanyl-tRNA synthetase 1 (AARS1) or AARS2 are used in the second pathway, which was recently identified. These enzymes directly transform lactate and ATP into lactate-AMP. Subsequently, the lactyl group is transferred to lysine residues on substrate proteins, resulting in lactylation modification (Ju et al., 2024; Mao et al., 2024; Zong et al., 2024). D-lactate is involved in non-enzymatic lactylation. The immediate substrate for this non-enzymatic activity is lactoylglutathione, which is created from methylglyoxal, a byproduct of glycolysis (Gaffney et al., 2020). When oxidative stress occurs, a drop in glutathione levels restricts glyoxalase 2 activity, which leads to an accumulation of S, D-lactoylglutathione, which can directly lactylate Lys through a non-enzymatic method (García-Giménez et al., 2025). It has been determined that L-lactylation is the main response to glycolysis and the Warburg effect, acting as a crucial epigenetic channel that connects transcriptional regulation with metabolic reprogramming (Zhang D. et al., 2025). However, it is yet unclear if D-lactylation can detect and react to dynamic changes in glycolytic flow in a comparable way. Interestingly, D-lactylation has not been found on histones in wild-type cells, indicating that it is not involved in nuclear processes and has a minimal impact on canonical histone modification (Wang and Zhang, 2025). More research is necessary to determine the particular molecular processes through which D-lactylation may contribute to the advancement of illness.

### 2.4 Key enzymes of lysine lactylation

**FIGURE 1:**
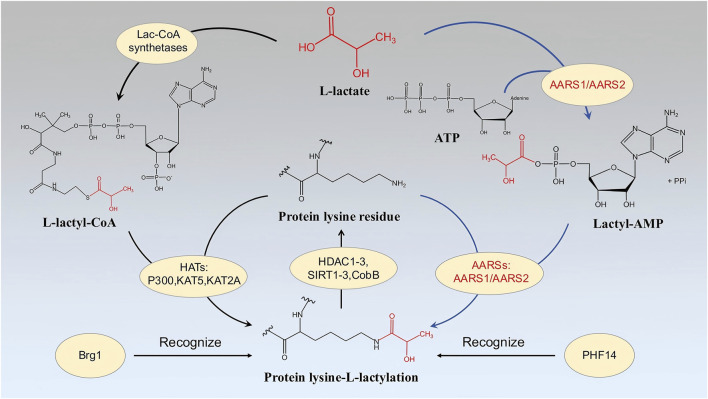
Enzymatic regulatory mechanism of protein L-lactylation. Key enzymes linked to protein L-lactylation are being identified and are commonly referred to as epigenetic tools. These tools include writers, enzymes that add specific acyl groups to protein-specific sites; erasers, a class of competent enzymes that remove acyl groups; and readers, a broad class of proteins with specialized structural domains that identify site-specific epigenetic markers. Abbreviations: Brg1: Brahma-related gene-1; HDAC: Histone deacetylase; HATs: Histone acetyltransferases; KAT5: Lysine acetyltransferase 5; KAT2A: Lysine acetyltransferase 2A; SIRT: Sirtuin; AARS1: Alanyl-tRNA synthetase 1; AARS2: Alanyl-tRNA synthetase 2; PHF14: PHD finger protein 14.

Lactylation mostly occurs at nucleophilic sites, like amino groups (-NH2), that interact with the associated functional groups. This degree of selectivity allows lactylation to specifically target particular proteins, altering their properties and functions (Moreno-Yruela et al., 2022). This suggests that lactylation is not caused by spontaneous chemical reactivity but rather by a sequence of reversible chemical processes that are catalyzed by enzymes. The involvement of writers, readers, and erasers is essential for lactylation modifications. Specifically, writers facilitate the insertion of lactyl groups, erasers are responsible for removing lactyl groups, and readers can recognize lactyl groups and interact with them (Figure 1) (Hu Y. et al., 2024).

#### 2.4.1 Writers

Numerous writers are involved in the lactylation process. The lactylation process is completed by the selective catalysis of the reactions between lactate and functional groups such as–OH or–NH2 groups by these writers. Recent reports have identified the acetyltransferases lysine acetyltransferase 5 (KAT5) and lysine acetyltransferase 2A (KAT2A) as potential writers involved in histone lactylation (Wang et al., 2022; Jia et al., 2023). KAT2A is often involved in tumor immune evasion, whereas KAT5 is associated with the autophagy pathway (Sun W. et al., 2023; Zhu R. et al., 2025). New research indicates that alanyl-tRNA synthetase 1/2 (AARS1/2) mediates a noncanonical lactylation process. Kla is directly catalyzed by AARS1/2 using free lactate as a substrate (Ju et al., 2024; Li H. et al., 2024; Zong et al., 2024). Among these lactyltransferases, p300 is notable. According to Liu et al., p300 promotes histone lactylation by forming a lactyltransferase complex with guanosine triphosphate-specific succinyl-CoA synthase in the nucleus (Liu R. et al., 2025). P300 is associated with multiple mechanisms. Enhanced lactyltransferase activity of p300 significantly elevates H3K9la levels at the tumor necrosis factor alpha (TNF-alpha) gene locus, thereby activating TNF-alpha transcription, amplifying TNF signaling, and promoting M1 microglial polarization (He et al., 2024). All the studies above indicate the significant role of writers, especially p300, in lactylation.

#### 2.4.2 Erasers

Histone deacetylase (HDAC) 1-3, sirtuin (SIRT) 1-3 and CobB are generally considered to act as erasers in lactylation. HDAC1, 2, and 3 have delactylase activity on both peptides and histones, but SIRT1, 2, and 3 exclusively have activity on histones (Moreno-Yruela et al., 2022). HDAC2 and HDAC3 have potent delactylase activity on peptides, with the activity of HDAC3 being several thousand times higher than that of SIRT2 (Zessin et al., 2022). According to research by Hong Weilong, the inhibition of the SIRT1/PGC-1α/LDHB axis in acetaminophen-induced liver injury leads to elevated levels of protein lactylation in the mitochondria of the liver (Hong et al., 2024). However, studies on erasers are relatively rare, and more research is urgently needed.

#### 2.4.3 Readers

Readers are able to identify and transduce lactylation signals by attaching to lactylated proteins. Recent research has indicated that bromodomain-containing proteins function as potential lactylation readers. Protein modules called bromodomains are composed of about 110 amino acids (Tian et al., 2025). The only protein found to be able to bind histone Kla peptides is the PHD-bromodomain of TRIM33 (Ren et al., 2025). Brahma-related gene-1 (Brg1), a bromine-containing protein, acts as a reader of histone lactylation. Brg1 specifically binds to H3K18la, leading to its accumulation on mesenchymal-epithelial transition-related gene promoters. Brg1 functions as a reader through the binding of the bromine domain to lactyl-lysine residues (Hu X. et al., 2024). Through a multivalent photoaffinity probe combined with a quantitative proteomics approach, double PHD fingers 2 has been confirmed to be the *bona fide* target of H3K14la, binding to H3K14la through its double PHD finger domain, with the key residue D274 directly interacting with Kla via hydrogen bonding (Zhai et al., 2024). In addition, plant homeodomain finger protein 14 has been characterized as a new lactylation reader that recognizes the long H3 N-terminal tail (Zheng et al., 2021; Tian et al., 2025). Despite these developments, little is known about lactylation-specific readers and the exact chemical processes by which they convey lactylation signals.

## 3 Regulation of cell death by lactylation

### 3.1 Regulation of apoptosis by lactylation

Apoptosis, which occurs when catabolic enzymes are activated downstream of mitochondrial cytochrome c release, is the most well-studied type of programmed cell death. This causes nuclear chromatin condensation or even nuclear fragmentation, membrane blebbing, and the quick breakdown of organelles and cellular structures (Gao et al., 2023). Apoptosis has important biological significance and involves complex molecular biological mechanisms. Specifically, too little cell death facilitates diseases of excessive proliferation, such as cancer, while too much cell death accelerates degenerative disorders, such as neurodegenerative diseases (Li K. et al., 2021; Newton et al., 2024). Lactylation has been found to regulate the process of apoptosis.

**FIGURE 2:**
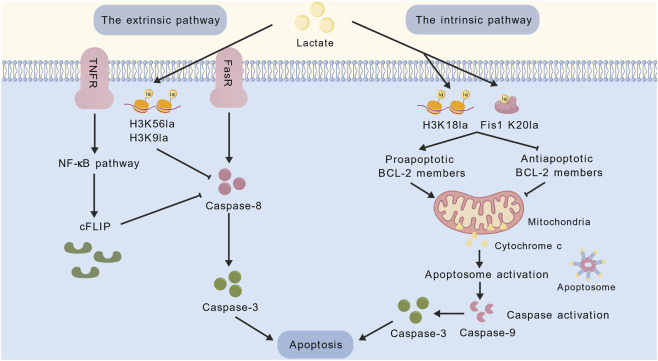
Regulation of apoptosis by lactylation. The extrinsic pathway is triggered mainly by the binding of death receptors to ligands, among which FasR tends to transmit apoptosis signals through caspase-8. TNFR tends to activate the NF-κB pathway to inhibit apoptosis, and histone lactylation affects apoptosis by regulating these two pathways. The intrinsic pathway involves the regulation of proapoptotic BCL-2 members and antiapoptotic BCL-2 family members, and histone lactylation regulates the release of mitochondrial cytochrome c by affecting the levels of these two types of proteins, which in turn affects apoptosis. Abbreviations: FasR: Fas receptor; TNFR: Tumor necrosis factor receptor; BCL-2: B-cell lymphoma 2; cFLIP: cellular FLICE-like inhibitory protein.

Mechanistically, apoptosis is activated through two major types of routes: extrinsic and intrinsic (Figure 2). The binding of death receptors to ligands is the main cause of the extrinsic route. Fas receptor (FASR) tends to transmit apoptosis signals through caspase-8, whereas tumor necrosis factor receptor (TNFR) tends to activate the NF-κB signaling pathway, which stimulates the production of cellular FLICE-like inhibitory protein (cFLIP) to inhibit apoptosis (Degterev et al., 2005). Increases in H3K56la and H3K9la have been reported to promote the occurrence and progression of hepatocellular carcinoma, whereas demethylzeylasteral promotes the expression of caspase-8 by reducing lactate production and inhibiting histone lactylation, thereby promoting the apoptosis of liver cancer stem cells (Pan et al., 2022).

Mitochondrial outer membrane permeabilization (MOMP), cytochrome c release from mitochondria, apoptosome formation with apoptotic protease-activating factor 1 (APAF1) and initiator caspase-9, and executioner caspase-3 activation are all part of the intrinsic pathway (Carneiro and El-Deiry, 2020). Proapoptotic B-cell lymphoma 2 (BCL-2) family members, such as BH3-only proteins, BCL-2-associated X protein (BAX), and BCL-2 antagonist killer 1 (BAK), positively regulate the release of cytochrome c from mitochondria, while antiapoptotic BCL-2 family members, such as BCL-W, BCL-2, and BCL-XL, negatively regulate it. The regulation of apoptosis by lactylation through intrinsic pathways has been demonstrated by several studies. In rat brain microvascular endothelial cells, H3K18la promotes the transcriptional activity of APAF1. Overexpression of APAF1 upregulates the protein expression of BAX and downregulates that of BCL-2 (Song et al., 2024). Moreover, in sepsis-induced acute kidney injury, lactate causes the lactylation of mitochondrial fission 1 protein (Fis1) lysine 20 (Fis1 K20la). An increase in Fis1 K20la expression promotes excessive mitochondrial fission, which subsequently inhibits the expression of BCL-2 and promotes the expression of BAK (An et al., 2023). BAX and BAK are central effectors of apoptosis. Proapoptotic proteins, such as BH3-only proteins, cause BAX and BAK to undergo conformational changes and oligomerization before forming a breach in the mitochondrial membrane that increases apoptosis and causes MOMP (Carneiro and El-Deiry, 2020). In addition, in neonatal rat cardiomyocytes, hypoxia/reoxygenation (H/R) induces neonatal rat cardiomyocyte injury and BCL-2 downregulation, ultimately leading to apoptosis. This effect can be lessened by overexpressing heat shock protein A12A (HSPA12A). HSPA12A maintains H3K56la in H/R cardiomyocytes, and HSPA12A protects cardiomyocytes from H/R-induced apoptosis through H3 lactylation (Yu et al., 2024). This finding highlights the subtle relationship between BCL-2 and lactylation in the regulation of apoptosis.

Lactylation plays a variety of roles in apoptosis, which highlights its intricate regulatory functions. Depending on the cellular environment, it can either promote or inhibit apoptosis. This duality has important implications for tumor progression and inflammatory responses, providing potential therapeutic targets for lactylation in a variety of diseases, especially cancer.

### 3.2 Regulation of autophagy by lactylation

Autophagy is a cellular mechanism whereby worn or unwanted parts are encased in double-coated autophagosomes (Ammanathan et al., 2020; Klionsky et al., 2021). The autophagosomes then mature and fuse with endosomes and finally with lysosomes. After fusion, lysosomal enzymes break down the encapsulated cytoplasmic material and inner membrane (Levine and Kroemer, 2019). This physiological process is essential for maintaining cellular homeostasis and organelle quality control and includes the recruitment of autophagy-related (ATG) proteins, the formation of autophagosomes, and autophagosome fusion with lysosomes (Ammanathan et al., 2020; Klionsky et al., 2021; Zhao et al., 2021). Lactylated core autophagy proteins can be important in both physiological and pathological processes, including ischemia, sepsis, neoplasia, and mitochondrial disorders (Sun W. et al., 2023).

Mammalian target of rapamycin (mTOR) hinders autophagic flow and inhibits cellular autophagy. In diabetic nephropathy, the lactate accumulation induced by the high-sugar environment in the presence of leucyl-tRNA synthetase 1 can activate the mTORC1 pathway and inhibit podocyte autophagy, leading to apoptosis and renal function impairment. In Alzheimer’s disease-transgenic model mice, a cigarette smoke-induced increase in lactate levels activates NOD-, LRR- and pyrin domain-containing protein 3 (NLRP3) transcription through H4 lysine 12 lactylation (H4K12la), which subsequently causes mTOR-mediated autophagy dysfunction in microglia. This promotes microglial activation and leads to Aβ plaque accumulation (Wang et al., 2024; Fan et al., 2025; Wang et al., 2025). One important regulatory element for the regulation of lysosomal and autophagy genes is transcription factor EB (TFEB) (Huang et al., 2024). Although TFEB overexpression promotes the breakdown of several autophagic payloads, the loss of TFEB impairs autophagosome formation and lysosome synthesis (Sardiello et al., 2009; Settembre et al., 2011; Settembre et al., 2013; Nezich et al., 2015). By protecting TFEB against E3 ubiquitin ligase WWP2-mediated ubiquitination and proteasomal destruction, lactate-induced lactylation of TFEB at lysine 91 increases lysosomal activity and autophagic flux. Human pancreatic ductal carcinoma samples showed increased lactylation of TFEB (Tan et al., 2022). By phosphorylating phosphatidylinositol to create phosphatidylinositol 3-phosphate, vacuolar sorting protein 34 (Vps34), a catalytic component of the class III PI3K complex, facilitates autophagy and endolysosomal trafficking. According to Jia et al., ULK1 increases lactate generation via phosphorylating lactate dehydrogenase A (LDHA), which in turn facilitates lactate-mediated Vps34 lactylation. By increasing lipid kinase activity, lactylated Vps34 increases endolysosomal degradation and autophagy. Lactate-mediated Vps34 lactylation stimulates autophagy, which is essential for muscle homeostasis and tumor progression in both physiological and pathological processes (Jia et al., 2023). Rubicon-like autophagy enhancer (RUBCNL/Pacer) promotes the maturation of autophagosomes. High levels of histone lactylation induced by aerobic glycolysis stimulate RUBCNL transcription in colorectal cancer cells, which promotes autophagy by stimulating autophagosome maturation and contributes to the development of colorectal cancer. Bevacizumab may further increase glycolysis in hypoxic cancer cells, which is followed by increased histone lactylation. Histone lactylation also transcriptionally upregulates RUBCNL expression, both of which help colorectal cancer cells survive and resist treatment (Li W. et al., 2024). H3K18la contributes to the development of Sjögren’s syndrome by directly controlling the autophagy signaling pathway in glandular epithelial cells (Chen et al., 2026). Therefore, the particular target proteins being altered may play a major role in the seemingly opposing effects of lactate-driven lactylation on autophagy. Lactylation of important autophagy activators increases their expression or stability, which stimulates autophagy. On the other hand, lactylation of autophagy-inhibitory proteins increases their inhibitory activity, which suppresses autophagy.

### 3.3 Regulation of pyroptosis by lactylation

**FIGURE 3:**
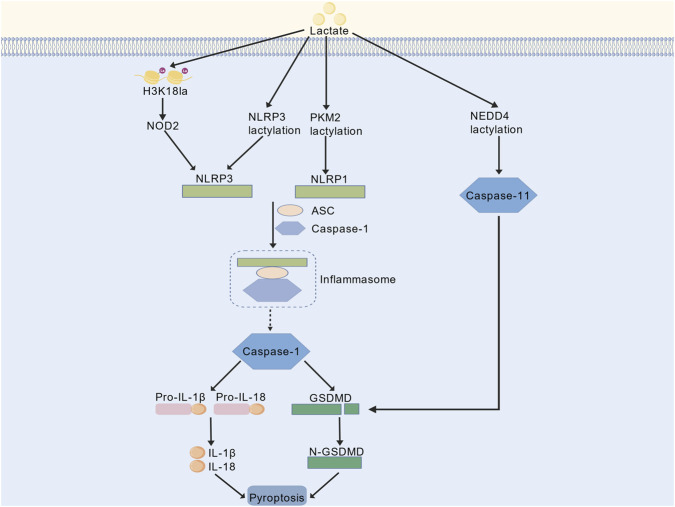
Regulation of pyroptosis by lactylation. The pyroptosis pathway is mainly divided into the canonical pathway, which is mediated by caspase-1, and the noncanonical pathway, which is mediated by caspase-4/5/11. In the canonical pathway, H3K18la-mediated NOD2 expression enhances bilirubin-induced pyroptosis. The lactylation of NLRP3 and pyruvate kinase M2 (PKM2) promotes the activation of the NLRP3 inflammasome and NLRP1 inflammasome, respectively, thereby facilitating the canonical pyroptosis pathway. In the noncanonical pathway, the lactylation of NEDD4 inhibits ubiquitination and increases the protein level of caspase-11, ultimately triggering pyroptosis. Abbreviations: NOD2: Nucleotide-binding oligomerization domain 2; NLRP3: NOD-, LRR- and pyrin domain-containing protein 3; NLRP1: NOD-, LRR- and pyrin domain-containing protein 1; PKM2: Pyruvate kinase M2; NEDD4: Neuronally expressed developmentally downregulated 4; GSDMD: Gasdermin D; ASC: Apoptosis-associated speck-like protein containing a caspase recruitment domain.

Because it is associated with innate and adaptive immunity, pyroptosis, a type of programmed cell death, is essential to host defense. Nevertheless, more investigation is required to ascertain whether pyroptosis is advantageous or harmful (Sun et al., 2021). In terms of morphology and pathophysiology, pyroptosis is a type of cell death that involves both apoptosis and necrosis. Rapid plasma membrane pore development, cell enlargement followed by osmotic necrosis, and the release of many cell contents and proinflammatory mediators are the hallmarks of pyroptosis. Pyroptosis can be divided into canonical pyroptosis and noncanonical pyroptosis (Figure 3) (Hara et al., 2025).

Canonical pyroptosis is mediated by caspase-1, which activates inflammasomes upon recognition of pathogen-associated or damage-associated molecular patterns, thereby cleaving gasdermin D (GSDMD) to release N-terminal fragments that form membrane pores, triggering cell lysis and the release of proinflammatory cytokines (Li Y. et al., 2021). Existing studies suggest that lactylation has a greater effect on the canonical pyroptosis pathway. Yao et al. demonstrated that LDHA-mediated histone lactylation might play a critical role in cerebral ischemia reperfusion injury. In Neuro-2a cells subjected to oxygen-glucose deprivation/reoxygenation, LDHA knockdown significantly reduces the levels of H3K18la expression and prevents pyroptosis. Mechanistically, H3K18la is enriched in the proximal promoter region of high mobility group box 1 (HMGB1), which further increases HMGB1 levels and causes pyroptosis (Yao and Li, 2023). Astrocytes stimulated with unconjugated bilirubin (UCB) showed elevated lactate levels and H3K18 lactylation. Inhibiting lactylation reduces UCB-driven pyroptosis and demonstrates that increased H3K18la expression upregulates the expression of nucleotide-binding oligomerization domain 2 (NOD2), which amplifies MAPK and NF-κB signaling to exacerbate neuroinflammation, neuronal loss, and neurological impairment in bilirubin-induced encephalopathy rats (Li J. et al., 2025). In addition, the regulation of pyroptosis by lactylation may provide ideas for the treatment of silicosis. Recent studies have demonstrated that aerobic glycolysis and the lactate produced by aerobic glycolysis might regulate the inflammasome activity of NLRP3 induced by crystalline silica and pyroptosis via protein lactylation, which is helpful for investigating silicosis pathogenesis (You et al., 2024). Moreover, the promotion of NLRP3 lactylation regulated by LDHA contributes to myocardial ischemia/reperfusion damage through the induction of cardiomyocyte pyroptosis. K245 is regarded as the lactylation site of NLRP3. The upregulation of lactylation plays a role in H/R-induced pyroptosis, in contrast to the effect of LDHA silencing (Fang et al., 2024). Additionally, mannose binds directly to PKM2, preventing its enzymatic activity and lowering the generation of lactate in bladder cancer. Owing to the increased acetylation and decreased lactylation caused by this decrease in lactate levels, PKM2 moves into the nucleus. The NF-κB pathway is triggered by nuclear PKM2, which results in pyroptosis that is dependent on NOD-, LRR- and pyrin domain-containing protein 1 (NLRP1)/caspase-1/GSDMD/IL-1β (Jin et al., 2025).

Noncanonical pyroptosis is activated by caspase-4/5 (in humans) or caspase-11 (in mice) through direct recognition of cytosolic lipopolysaccharide. These caspases cleave GSDMD to create membrane holes and promote the release of proinflammatory cytokines by activating caspase-1, resulting in cell death and inflammatory responses (Downs et al., 2020). Studies have shown that lactate directly adds lactyl groups to the protein, causing neuronally expressed developmentally downregulated 4 (NEDD4) to become lactylated. This process, which is reliant on both endogenous and exogenous lactate levels and p300/SIRT1 activity, causes NEDD4 binding to caspase-11 to be inhibited, which in turn prevents ubiquitination and increases caspase-11 protein levels. This results in increased noncanonical pyroptosis and exacerbates secondary damage from acetaminophen-induced liver injury (Li Q. et al., 2024).

### 3.4 Regulation of ferroptosis by lactylation

One form of nonapoptotic cell death that is dependent on iron is ferroptosis. By altering iron homeostasis, a key factor in this cell death process, lactate also controls ferroptosis sensitivity (Fang et al., 2023). Ferric iron is transported into cells by transferrin, which binds to the transferrin receptor (TFRC) to facilitate cellular iron uptake (Ru et al., 2024). It has been demonstrated that lactylation of lysine-specific demethylase 1 (LSD1) increases its association with FosL1, which suppresses ferroptosis and represses TFRC transcription in BRAFi/MEKi-resistant melanoma (Li A. et al., 2025). Ferroptosis regulation depends not only on iron transport but also on iron storage. By sequestering excess iron, ferritin heavy chain 1 (FTH1), a crucial ferritin component, acts as a negative regulator of ferroptosis (Fang et al., 2021). ChIP experiments showed that zinc finger protein 64 (ZFP64) directly binds to the FTH1 and guanosine triphosphate cyclohydrolase 1 (GCH1) promoters in triple-negative breast cancer cell lines, increasing their transcription. Mechanistically, lactate from cancer-associated fibroblasts causes H3K18la alteration, which increases ZFP64 expression and then inhibits ferroptosis via GCH1-mediated lipid peroxidation suppression and FTH1-mediated Fe^2+^ depletion (Zhang K. et al., 2025). Additionally, under sublethal heat stress, lactate-driven H3K18la change has been shown to enhance NFS1 cysteine desulfurase (NFS1) transcriptional activity in hepatocellular carcinoma cells (Huang et al., 2025). Crucially, it has been demonstrated that NFS1, an essential enzyme for the production of Fe-S clusters, limits the release of free iron, protecting cells from ferroptosis (Huang et al., 2025). All of these findings point to lactylation as a crucial iron metabolism regulator that helps cancer cells avoid ferroptosis.

Recent research has revealed a wider role for lactate-derived lactylation in regulating lipid peroxidation and antioxidant defense, two crucial mechanisms influencing ferroptosis sensitivity, in addition to iron metabolism. Elevated H3K18la at the absent in melanoma 2 (AIM2) promoter region was identified by Wu et al. as the cause of the pathological increase of glycolysis in lung cancer cells (Wu et al., 2025). In particular, it has been shown that H3K18la-mediated overexpression of AIM2 attenuates ferroptosis via the STAT5b/ACSL4 signaling axis (Wu et al., 2025). By reducing lipid peroxidation, glutamate-cysteine ligase (GCLC), an essential enzyme for glutathione production, inhibits ferroptosis (Deng et al., 2025). Lactylation controls GCLC expression at several levels, according to recent data. In particular, it has been demonstrated that H4K12la promotes GCLC transcription in colorectal cancer stem cells (Deng et al., 2025), whereas lactate-mediated lactylation of NOP2/Sun RNA methyltransferase family member 2 (NSUN2) at K508 boosts its ability to stabilize GCLC mRNA, giving gastric cancer cells resistance to ferroptosis (Niu et al., 2025). Additionally, by converting CoQ to CoQH2, ferroptosis suppressor protein 1 (FSP1) creates a different ferroptosis defensive mechanism (Li et al., 2023). According to Yang et al., HDAC inhibition lowers HDAC1 lactylation, which lessens m^6^A modification on FSP1 mRNA. This decrease lowers FSP1 protein levels and makes cancer cells more vulnerable to ferroptosis by encouraging degradation of FSP1 mRNA (Yang et al., 2025). In order to decrease ferroptosis and increase tumor cell survival, lactate-derived lactylation of both histone and non-histone proteins may alter important ferroptosis regulators while concurrently regulating lipid peroxidation and antioxidant pathways.

### 3.5 Regulation of cuproptosis by lactylation

In contrast to ferroptosis, cuproptosis is a metal-driven cell death modality that depends on mitochondrial respiration and copper. The instability of Fe-S cluster proteins and lipoylated protein aggregation brought on by copper buildup cause cuproptosis, which ultimately results in proteotoxic stress and cell death (Xie et al., 2023). Cuproptosis is associated with increased mitochondrial respiration rates, ferrodoxin-1 (FDX1) expression levels, and copper availability (Chen et al., 2022; Tsvetkov et al., 2022). New research shows that lactylation is crucial for controlling the machinery involved in cuproptosis. FDX1 is the primary regulator of cuproptosis, which converts Cu(II) to Cu(I) and promotes protein lipoylation and the consequent aggregation of dihydrolipoamide S-acetyltransferase (DLAT) in mitochondria (Sun et al., 2025). In gastric cancer, copper stimulates methyltransferase-like 16 (METTL16) lactylation, which stabilizes FDX1 and causes cuproptosis. A viable approach to cancer treatment is to target cuproptosis and lactyl-METTL16 (Sun L. et al., 2023). According to Lin et al., lactate-induced lactylation of nudix hydrolase 21 (NUDT21) drives transcriptomic reprogramming through alternative polyadenylation modulation. Lactylation of NUDT21 improves its interaction with cleavage and polyadenylation specificity factor 6, which promotes the development of the CFIm complex and lengthens the 3′ untranslated region (UTR) of FDX1. In esophageal squamous cell carcinoma, extension of the FDX1 3′ UTR reduces the protein output, providing conferring resistance to cuproptosis (Lin et al., 2025). In colorectal cancer, N-acetyltransferase 10 (NAT10) has been reported to promote cuproptosis via regulating the N4-acetylcytidine alteration of DLAT mRNA stability. Additionally, lactylation of NAT10 at K426 was found to increase the catalytic activity of NAT10. Conversely, SIRT1 inhibits cuproptosis via mediating the delactylation of NAT10-K426 (Yang et al., 2026). In conclusion, lactylation is a crucial regulatory mechanism of cuproptosis, and the chemical identity of the lactylated substrates determines whether lactylation promotes or inhibits the process. The precise circumstances under which lactylation stimulates or inhibits cuproptosis, however, need more research due to the paucity of literature on the subject.

## 4 The crosstalk between lactylation and other PTMs on cell death

When lactylation takes place on the ε-amino group of lysine residues, it competes with other PTMs such acetylation, ubiquitination, methylation, succinylation, and crotonylation that also target the lysine ε-amino group. Furthermore, even though these modifications are not principally linked to lysine, lactylation shows intricate interactions with a variety of other PTMs, including phosphorylation. Since the connections between lactylation and other PTMs have not been thoroughly studied, especially in the fileds of cell death, we concentrate on the interactions between lactylation and acetylation, ubiquitination, and phosphorylation.

### 4.1 Lactylation and acetylation

The writers and erasers of Kla that have been identified thus far have a lot in common with those engaged in acetylation. But the substrates for the two modifications are different. Lactylation mainly depends on lactyl-CoA, with lactate playing a minor role, whereas acetylation depends on acetyl-CoA. As a result, the availability of their corresponding substrates and the enzymes involved in sugar metabolism dynamically control these alterations. For example, pyruvate dehydrogenase E1 component subunit alpha 1 was rendered inactive by hyperacetylation, which stopped pyruvate from being transformed into acetyl-CoA. As an outcome, lactate accumulated, which in turn increased the Kla of proteins like Fis1 and finally caused apoptosis (An et al., 2023). Kla and acetylation may fight for the same lysine sites since they both act on lysine residues. For instance, p53’s K120la can block its K120ac, impeding its ability to activate transcription and stopping tumor cells from apoptosis (Zong et al., 2024). Additionally, Kla may control cell death through modifying common enzymes like HDACs. In particular, HDAC1’s Kla may hinder its deacetylation function, which would lower histone acetylation levels and lessen ferroptosis sensitivity (Yang et al., 2025). In conclusion, substrate competition and the control of common enzymes maintain a delicate balance between Kla and acetylation, which modulates cell death processes.

### 4.2 Lactylation and ubiquitination

A common PTM, ubiquitination controls protein stability via the ubiquitin-proteasome system and is vital for cell death. According to recent research, Kla contributes to the control of cell death by competitive inhibition, direct influence on the activity of ubiquitination enzymes, and modulation of their expression. For example, histone Kla increased ubiquitin-specific peptidase 39 expression, which deubiquitinated phosphoglycerate kinase 1 and further inhibited apoptosis (Wei et al., 2024). As consequently, Kla controls ubiquitin enzymes by directly affecting their activities as well as indirectly modifying their expression. In some instances, Kla’s competitive suppression of ubiquitination may cause the ubiquitin enzyme to lose its capacity to regulate particular target proteins, which could alter the stability of those proteins. For instance, discoidin, CUB, and LCCL domain-containing type I (DCBLD1)’s K172la directly prevented its ubiquitination, which decreased DCBLD1 degradation and hindered apoptosis (Meng et al., 2024). By modifying enzyme systems and substrate competition, Kla and ubiquitination interact to precisely control cell death and impact one another.

### 4.3 Lactylation and phosphorylation

Phosphate groups are transferred to substrates during phosphorylation, which is mostly carried out by kinase enzymes and is essential for controlling signal transduction and enzyme function. The interaction between phosphorylation and Kla has important ramifications for the investigation and management of a number of illnesses, such as cancer and neurological conditions. Phosphorylation functions as an upstream regulator of Kla. For instance, phosphorylation activates LDHA, which catalyzes the synthesis of lactate, hence increasing Kla (Jia et al., 2023; Liu et al., 2024). These modifications also play a role in controlling cell death. However, phosphorylation and Kla interact in a complicated way, showing both inhibitory and synergistic effects on substrate changes. For example, it was discovered that Kla of the transcription factor Sox10, which is connected to pyroptosis, depends on its previous phosphorylation (Xu et al., 2023). On the other hand, Kla of AMPKα inhibited its phosphorylation, which increased apoptosis and cellular senescence (Zhang et al., 2024). The structural features of the active sites of particular substrates may be responsible for this varied regulation. In general, phosphorylation and Kla interact at several levels, and comprehending the processes behind this connection is essential to understanding how cellular metabolic and signaling pathways are coupled.

## 5 Therapeutic targeting of lactylation

New regulatory mechanisms at the intersection of lactylation and various types of cell death offer insights into potential therapeutic targets. Controlling lactylation provides new treatment options for illnesses, including cancer and neurological diseases, that are characterized by dysregulated cell death. Therefore, targeting lactylation is a useful strategy for enhancing disease pathogenesis.

Lactyltransferases are enzyme mediators of lactylation, and their identification and characterization are still being researched. Directly regulating lactylation-dependent gene expression without changing systemic lactate levels is possible by targeting these enzymes. Several small-molecule medications that were first created as inhibitors of acetyltransferase have shown cross-reactivity with lactylation pathways, indicating that they may be new therapeutic targets.

Among these, the well-known p300/CREB acetyltransferase activity inhibitor C646 also demonstrates lactylation-inhibiting characteristics. This could lead to new research avenues and offer important insights into the interactions and crosstalk between various epigenetic changes. Furthermore, A-485, a p300 writer antagonist, efficiently prevents protein lactylation and enhances functional recovery in models of ischemic stroke (Xiong et al., 2024).

Many inhibitors have been developed with regard to lactylation erasers, namely, HDACs, such as trichostatin A, MS275, nicotinamide, RGFP966, and RGFP109. Further investigation is necessary to ascertain the precise effects of these drugs on delactylation processes even though they have shown promise in reducing inflammation under hypoxia through nonlactylation pathways (Yang et al., 2016; Liao et al., 2020; Song et al., 2025). Additionally, Honokiol, an activator of SIRT3, has demonstrated antitumor efficacy in hepatocellular carcinoma by directly activating delactylation enzymes (Jin et al., 2023). To assess the safety and effectiveness of these promising medicinal medicines in the treatment of diseases associated with lactylation, more clinical trials are needed.

## 6 Conclusion and future perspectives

**FIGURE 4:**
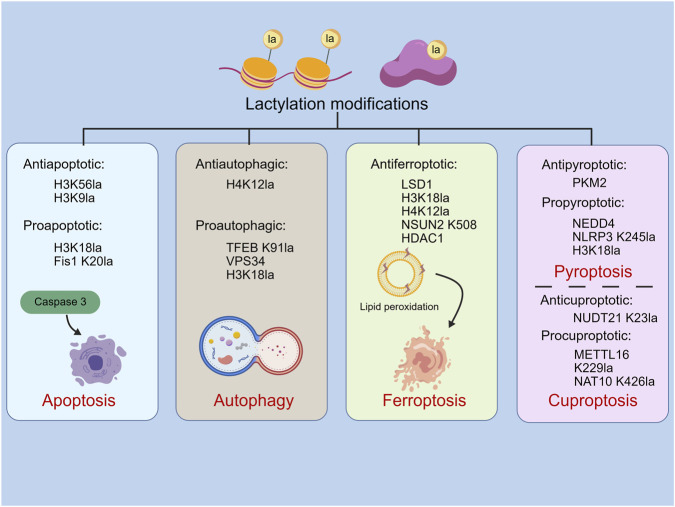
Regulatory roles of lactylation in various forms of cell death. Lactylation plays a dual in multiple types of cell death, including apoptosis, autophagy, pyroptosis, ferroptosis, and cuproptosis. Key gene expression and protein functions involved in cell death pathways can be modulated by lactate-derived lactylation. Abbreviations: Fis1 K20la: Fission 1 protein lysine 20; NLRP3: NOD-, LRR- and pyrin domain-containing protein 3; TFEB: Transcription factor EB; LSD1: Lysine-specific demethylase 1; Vps34: Vacuolar sorting protein 34; PKM2: Pyruvate kinase M2; NEDD4: Neuronally expressed developmentally downregulated 4; NSUN2: NOP2/Sun RNA methyltransferase family member 2; HDAC1: Histone deacetylase 1; METTL16: Methyltransferase-like 16; NUDT21: Nudix hydrolase 21; NAT10: N-acetyltransferase 10.

Lactylation affects cellular life processes by controlling gene expression, modifying the activity and stability of proteins, altering protein localization, and influencing signal transduction pathways. Lactylation plays a critical role in the regulation of cell death, such as apoptosis, autophagy, ferroptosis, pyroptosis, and cuproptosis (Table 1). As previously mentioned, depending on the type of disease and the cellular environment, lactylation can either promote or inhibit cell death (Figure 4). Additionally, lactylation may exert its effects through competition and synergy with other PTMs. Cellular metabolism and lactylation levels are intimately related; therefore, studies on the relationship between lactylation and cell death has expanded our knowledge of how metabolism and disease progression are related.

**TABLE 1:** Lactylation modifications associated with cell death.

Cell death	Site	Target cell	Disease	Function
Apoptosis	H3K56, H3K9	Liver cancer stem cell	Hepatocellular carcinoma	Inhibits the expression of caspase-8 (Pan et al., 2022)
	H3K18	Rat brain microvascular endothelial cell	Cerebral ischemic stroke	Promotes the expression of APAF-1 (Song et al., 2024)
	H3K56	Neonatal rat cardiac myocyte	Acute myocardial infarction	Protects cardiomyocytes from Ischemia-reperfusion injury (Yu et al., 2024)
	Fis1 K20la	Human kidney-2 cell	Sepsis-induced acute kidney injury	Promotes excessive mitochondrial fission (An et al., 2023)
Autophagy	H4K12	Microglia	Alzheimer’s disease	Actives NLRP3 protein (Wang et al., 2024; Fan et al., 2025; Wang et al., 2025)
	TFEB	Macrophage	Bacterial infection	Increases lysosomal activity and autophagy flux (Tan et al., 2022)
	PIK3C3/Vps34	Muscle cell, cancer cell	Cancer	Activates Vps34 lipid kinase activity (Jia et al., 2023)
	H3K18	Hypoxic cancer cell	Colorectal Cancer	Activates RUBCNL transcription (Li et al., 2024c)
	H3K18	Glandular epithelial cells	Sjögren’s syndrome	Controls the autophagy signaling pathway (Chen et al., 2026)
Pyroptosis	H3K18	Neuro-2a	Cerebral Ischemia/Reperfusion	Facilitates the expression of HMGB1 (Yao and Li, 2023)
	H3K18	Astrocyte	Bilirubin encephalopathy	Promotes the expression of NOD2 (Li et al., 2025b)
	NLRP3 K245	Macrophage	Silicosis	Promotes the activation of inflammasomes (You et al., 2024)
	PKM2	Bladder cancer cell	Bladder cancer	Triggers the NF-κB pathway (Jin et al., 2025)
	NEDD4	Macrophage	Liver injury	Raises caspase-11 protein levels (Li et al., 2024b)
Ferroptosis	LSD1	Melanoma cells	Melanoma	Increases the association with FosL1 (Li et al., 2025a)
	H3K18la	triple-negative breast cancer cell	triple-negative breast cancer	Increases ZFP64 expression (Zhang et al., 2025b)
	H3K18la	hepatocellular carcinoma cell	hepatocellular carcinoma	Enhance NFS1 transcriptional activity (Huang et al., 2025)
	H3K18la	Lung cancer cell	Lung cancer	Promotes the expression of AIM2 (Wu et al., 2025)
	H4K12la	Colorectal cancer stem cell	Colorectal cancer	Promotes GCLC transcription (Deng et al., 2025)
	NSUN2 K508	Gastric cancer cell	Gastric cancer	Enhances the stabilization of GCLC mRNA (Niu et al., 2025)
	HDAC1	Colorectal cancer cell	Colorectal cancer	Lessens m^6^A modification on FSP1 mRNA (Yang et al., 2025)
Cuproptosis	METTL16	Gastric cancer cell	Gastric cancer	Enhances m^6^A modification of FDX1 mRNA (Sun et al., 2023a)
	NUDT21	Esophageal squamous cell carcinoma cell	Esophageal squamous cell carcinoma	Lengthens the 3′ UTR of FDX1 (Lin et al., 2025)
	NAT10 K426	Colorectal cancer cell	Colorectal cancer	Increases the catalytic activity of NAT10 (Yang et al., 2026)

Abbreviations: APAF-1: Apoptotic protease-activating factor 1; Fis1 K20la: Fission 1 protein lysine 20; NLRP3: NOD-, LRR- and pyrin domain-containing protein 3; TFEB: Transcription factor EB; PIK3C3: Phosphoinositide-3-kinase class 3; Vps34: Vacuolar sorting protein 34; RUBCNL: Rubicon like autophagy enhancer; HMGB1: High mobility group box 1; NOD2: Nucleotide-binding oligomerization domain 2; PKM2: Pyruvate kinase M2; NEDD4: Neuronally expressed developmentally downregulated 4; LSD1: Lysine-specific demethylase 1; FosL1: Fos-related antigen 1; ZFP64: Zinc finger protein 64; NFS1: NFS1 cysteine desulfurase; AIM2: Absent in melanoma 2; GCLC: Glutamate-cysteine ligase; NSUN2: NOP2/Sun RNA methyltransferase family member 2; HDAC1: Histone deacetylase 1; m6A: N6-methyladenosine; FSP1: ferroptosis suppressor protein 1; METTL16: Methyltransferase-like 16; FDX1: Ferrodoxin-1; NUDT21: Nudix hydrolase 21; NAT10: N-acetyltransferase 10.

Despite recent progress, many unknowns remain in the study of lactylation. First, the relationship between lactylation levels and cell death needs further exploration. Second, more potential lactylation sites need to be identified, including the possibility of modifications occurring on residues other than lysine. Third, the study of lactylation readers remains largely unexplored, and further research is needed to elucidate more types and functions of writers and erasers. Fourth, the specific pathways through which histone and nonhistone lactylation modifications regulate cell death have yet to be elucidated. The interaction between lactylation and other PTMs (such as methylation and phosphorylation) and its impact on cell death are also important areas that require further investigation.

Lactylation provides a new entry point for intervening in cell death, with significant clinical application potential. Investigating the association between lactylation and cell death may help identify novel biomarkers that are indicative of disease states. These biomarkers could be utilized for early diagnosis, therapeutic efficacy evaluation, and prognostic assessment. Understanding the underlying mechanisms of lactylation across different modes of cell death could facilitate the discovery of new therapy targets and the development of innovative intervention strategies. Clinically, modification-targeted drugs, such as KAT and HDAC inhibitors, are frequently used to treat cancer (Zhao et al., 2023). Research into lactylation mechanisms may also contribute to overcoming drug resistance in cancer therapy.

To comprehend their regulatory roles in cell death and to elucidate their mechanisms in various diseases, lactylation sites must be accurately identified. At present, mass spectrometry remains the most widely used method for site identification, offering high throughput and precision; but its use is constrained by high prices and time requirements. To overcome these challenges, a series of AI-based computational models have been continuously developed, leading to significant advances in Kla site prediction and steadily improving generalizability and accuracy. In the future, with advancements in sequencing technologies and artificial intelligence, more systematic and accurate high-throughput sequencing methods, proteomics approaches, and bioinformatics tools are expected to emerge and be applied to the identification and quantification of lactylation modifications.

Research on the relationship between lactylation and cell death has enhanced our understanding of cell biology and medicine and offers promising opportunities for clinical applications. However, the mechanisms and biological functions of lactylation in regulating cell death still require more comprehensive and in-depth research in the future.
